# MicroRNAs in mouse development and disease

**DOI:** 10.1016/j.semcdb.2010.02.004

**Published:** 2010-09

**Authors:** Morag A. Lewis, Karen P. Steel

**Affiliations:** Wellcome Trust Sanger Institute, Wellcome Trust Genome Campus, Hinxton, Cambridge CB10 1SA, UK

**Keywords:** MicroRNA, Mouse mutants, Disease models, Disease mechanisms

## Abstract

MicroRNAs, small non-coding RNAs which act as repressors of target genes, were discovered in 1993, and since then have been shown to play important roles in the development of numerous systems. Consistent with this role, they are also implicated in the pathogenesis of multiple diseases. Here we review the involvement of microRNAs in mouse development and disease, with particular reference to deafness as an example.

## 1 Introduction

MicroRNAs are small non-coding RNAs which negatively regulate specific target genes by mRNA degradation or translational repression, and they are numerous throughout the genomes of both animals and plants. They demonstrate a range of spatial and temporal expression patterns, and have proven to play multiple roles in a variety of processes. The first microRNA identified, the *lin-4* locus in the nematode worm *Caenorhabditis elegans*, was found because mutations cause abnormal cell division and proliferation, and also affect the timing of cell division and development in larvae [1]. When the gene responsible was cloned, rather than a protein-coding gene, it was found to code for a small RNA that bound to and downregulated the messenger RNA of another gene named *lin-14*, which *lin-4* was known to negatively regulate [2]. It was 7 years before the second small regulatory RNA was found: *let-7*, also in *C. elegans*. Like *lin-4*, *let-7* controls cell fate and timing; loss of the gene causes defects during moulting where cells failed to achieve adult identities. Overexpression of *let-7* has the opposite effect; cells differentiate too early [3]. Homologues of *let-7* were found in multiple lineages, from vertebrates to arthropods, and expression of *let-7* in zebrafish and fruit fly was limited to late stages in development. It was therefore suggested that these ‘small temporal RNAs’ might play a role in the timing of developmental transitions [4].

It is now known that microRNAs have far more roles than just timing developmental transitions, and they are present in multiple species, including plants, animals and even an alga, implying that the regulation of genes by RNA silencing is an ancient mechanism [5]. Like *let-7*, homologues of some microRNA families can be found in deuterostomes and protostomes, while some are more specific, such as *Mir196*, which is limited to vertebrate species, having no close orthologues in invertebrates, although it is related distantly to *let-7* [6]. *Mir196* is found in the lamprey, a jawless fish, which means it must have arisen before the divergence of the agnathans and the gnathostomes, a very early event in the vertebrate lineage [7]. miR-196a cleaves the transcript of the homeobox gene *Hoxb8* in mouse embryos, important in specifying regional identity in the embryo [8–10].

## 2 Expression patterns of microRNAs and their targets

Many more microRNAs are known to be expressed throughout development, so much so that it has been suggested that each cell type has its own microRNA expression profile, and dynamic, localised expression of any gene during the development of a specific system implies a function for that gene [11]. For example, microRNA expression has been shown to vary dynamically in the brain both before and after birth, indicating a requirement for different microRNAs at different time points [12]. Likewise, the microRNA *Mir133* is known to be specifically expressed in the mouse embryonic heart and skeletal muscle from E12 onwards, implying a role in mid-gestation development of these tissues [13].

The microRNA expression profile is only one part of the jigsaw puzzle; a microRNA may be present ubiquitously, but if its target gene is only expressed in a small subset of cells, then that is where that microRNA will exert its effects. The importance of expression patterns of both the microRNAs and their targets can be illustrated by miR-1, which is expressed in embryonic heart and skeletal muscle. Hdac4 is a repressor of muscle differentiation, and miR-1 is thought to target Hdac4 in muscles and thus promote muscle differentiation [14]. In the heart, however, miR-1 is required to regulate ventricular cardiomyocytes through repressing the cardiac transcription factor Hand2; *Mir1* overexpression results in heart failure at about E13.5, due to a failure in proliferation [15].

## 3 MicroRNAs have many targets

The identification of target genes is, of course, very important for understanding how microRNAs operate. So far, many of the microRNA studies that have been carried out have concentrated on the effect of the particular microRNA on a single gene or just a few genes. For example, results from experimental manipulation of expression of miR-150 and its most likely target gene, *Myb*, together with the observation of adverse effects of the systemic knockout of miR-150 on a single cell type only, the mature lymphocyte, suggest that the main role of this microRNA may be mediated through interaction with a single target gene in a single cell type [16]. Similarly, genetic manipulation of both miR-223 and its most likely target gene, *Mef2c*, suggested that this microRNA acted primarily through its interaction with *Mef2c* in early myeloid progenitors [17].

**Fig. 1:**
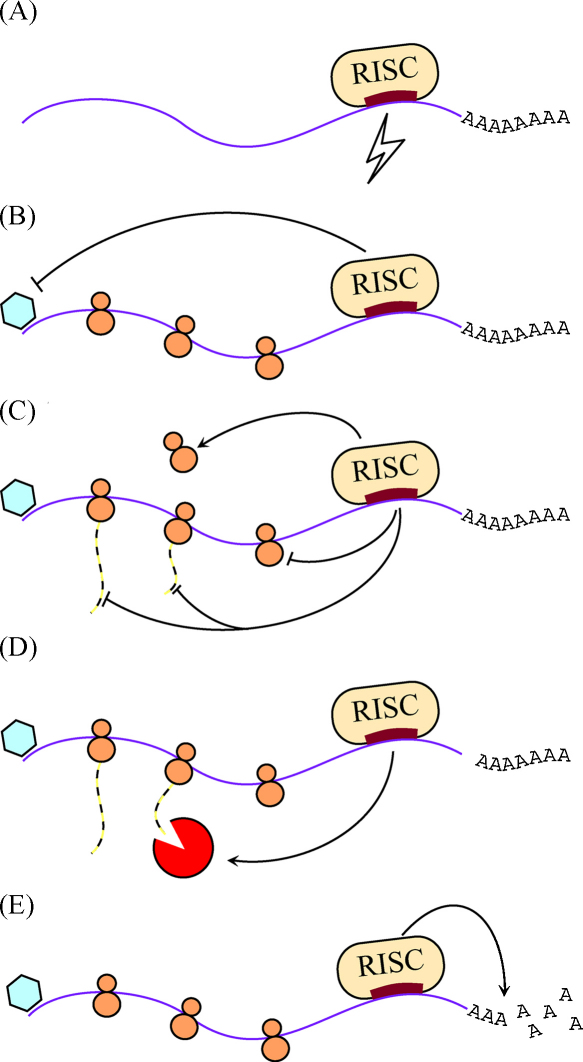
Cartoon showing putative mechanisms of microRNA repression. The microRNA is represented by a thick red block over the violet line, which is the target mRNA. The blue hexagon represents the translation initiation complex and the orange circles ribosomes. (A) Perfect binding of miRNA to target mRNA and cleavage of mRNA followed by degradation. (B) Inhibition of translation initiation. (C) Inhibition of elongation/termination and promotion of ribosome drop-off. (D) Recruitment of a proteolytic enzyme to degrade emerging polypeptides. (E) Deadenylation, followed by degradation, of the mRNA [78,79].

However, bioinformatic analyses predict hundreds of targets for mammalian microRNAs, and it seems likely that many are genuine targets [18]. This is backed up by multiple studies *in vivo* and *in vitro*. For example, mice null for *Mir223* show derepression of a wide range of both proteins and mRNAs, which in turn indicates that microRNAs in animals may operate by the degradation of messenger RNA, prevention of protein translation or a combination of both [19] (Fig. 1). A second example is the mutant lacking *Mir155*, which is immunodeficient and displays lung and airway remodelling and shows a wide range of genes upregulated in their Th1 and Th2 cells, many of which are predicted targets of miR-155 [20]. Thirdly, miR-1 and miR-124, when transfected into human cells, cause downregulation of about 100 messages each [21]. A fourth example is the transfection of several human microRNAs into HeLa cells, each of which led to downregulation of hundreds of mRNAs together with modest reduction in protein levels [22]. Finally, our own analysis of the impact of a mutation affecting miR-96 on mRNA expression in the inner ear indicates that many hundreds of transcripts are affected (see later) [23].

It has been suggested that microRNAs exert both absolute and fine-tuned control of gene expression, adjusting levels of transcripts to give either complete or simply decreased repression. Such ‘fine-tuning’ microRNAs are likely to be harder to identify than those operating by ‘switching off’ a gene, since loss of function of any one of them would be predicted to have subtle effects that are hard to characterise and study [24].

## 4 MicroRNAs in early mouse development and cancer

In the mouse zygote, maternally inherited microRNAs are abundant, with a dynamic expression profile; some are downgraded by as much as 95% between the one- and two-cell stages. Zygotic transcription of microRNAs begins from the two-cell stage. Oocytes lacking *Dicer*, a gene required for microRNA processing and essential for mouse development [25], lack maternal microRNAs and fail to pass the first cell division [26,27]. One of the many microRNAs expressed from the two-cell stage is *Mir125a*, an orthologue of *C. elegans lin-4*. Its expression increases between the two-cell and blastocyst stages, and it is thought that it may have a role in controlling early embryonic timing through regulation of the gene *Ped* [28]. *Mir93*, a member of the miR-17 family, is specifically expressed in the trophoectoderm and future primitive endoderm, in a pattern complementary to that of the gene *Stat3*, which is important for early development [29,30]. miR-93 has been shown to bind to and downregulate *Stat3* mRNA during ES cell differentiation [29].

It is common for genes controlling developmental processes such as cell proliferation and differentiation to be associated with cancer, and many microRNAs are implicated in tumour development and progression, with a significant number of miRNAs located close to tumour susceptibility loci in mice [31]. The miR-17–92 cluster, for example, is required for proper development of the heart and lung, and also that of foetal and adult B cells, possibly through regulation of the pro-apoptotic gene *Bim* [32,33]. It was first known, however, for its role in cancer; overexpression of part of the cluster in a mouse B-cell lymphoma model causes acceleration of tumour development [34].

## 5 MicroRNA expression profiles in disease

Comparison of microRNA expression profiles in diseased and healthy tissues has shown the diseased state to be associated with changes in the expression levels of many different miRNAs, and we give just a few examples. The miRNA expression profile of the retinas of mice with retinitis pigmentosa showed significant differences compared with wildtype retinas, implying that microRNAs could play a role in retinal disease [35]. MicroRNA expression in the diaphragm of a dystrophin-deficient mouse, a model for Duchenne muscular dystrophy, revealed a dramatic increase in expression of the muscle-specific microRNA *Mir206* [36], while skeletal muscle hypertrophy induced by functional overloading of the plantaris muscle results in downregulation of miR-1 and miR-133a, which are also muscle-specific miRNAs [37]. In a third example, mice subjected to thoracic aortic banding, which in wildtype mice induces cardiac hypertrophy, revealed over 100 miRNAs with differential expression compared to control mice [38]. The most upregulated gene in this condition was *Mir21*. When miR-21 was knocked down using an antisense approach *in vitro*, cardiomyocyte hypertrophy was reduced, suggesting it has a key role in the mechanism of cardiac hypertrophy [38].

## 6 Mouse knockouts demonstrate the importance of specific microRNAs

Knockouts are of course very important for studying the role of a gene in pathogenesis. For example, knockouts have demonstrated that several microRNAs are required for heart development and function. Mice null for both miR-133a-1 and miR-133a-2 have larger ventricular chambers, and thinner ventricular walls than controls, and proved to have aberrant cardiomyocyte proliferation and apoptosis [39]. miR-208 knockout mice display a reduced hypertrophic response to thoracic aortic banding, and this microRNA is thought to regulate stress-dependent gene expression in the heart [40] and is required for normal cardiac conduction [41]. Mice lacking miR-1-2 have defects in cardiac morphogenesis and conduction [42]. miR-17–92 knockout mutants died at birth with a ventricular septal defect as well as hypoplastic lungs [33].

Targeted microRNA knockouts have not been limited to those microRNAs involved in heart development and function. For example, *Dnmos3*, a non-coding RNA transcript which is processed into three microRNAs, miR-199a, miR-199a* and miR-214, is expressed from E9.5 in multiple locations and knockouts show reduced viability and skeletal defects, with shortened heads, osteopenia, and defects in the neural arch and spinous process [43]. Mice null for miR-143 and miR-145 demonstrate dedifferentiation of vascular smooth muscle cells, implying a role for those microRNAs in maturation and differentiation of these cells [44,45]. Intriguingly, miR-143 and miR-145 have been found to be deregulated in aortic aneurysms in humans [46]. *Mir155* knockout mice display enteric inflammation and changes in their lungs similar to lung fibrosis, suggesting a role for miR-155 in the immune system. Immunized *Mir155* were not protected against *Salmonella typhimurium*, indicating a lack of protective immunity. Indeed, B and T cell responses are reduced in the *Mir155* knockout, and miR-155 is implicated in Th1 and Th2 cell development and dendritic cell function [20,47]. Mice lacking miR-375 show abnormal glucose homeostasis and pancreatic α and β cell numbers [48].

Many microRNAs are located in the introns of coding genes. Almost 200 of these genes were experimentally disrupted before it was appreciated that they harboured a microRNA [49]. In at least one case, the knockout of the *Egfl7* locus, the vascular phenotype and extensive embryonic lethality observed in the mutants has been shown to result from the concomitant disruption of miR-126 rather than inactivation of the *Egfl7* gene, by selectively inactivating either miR-126 or *Egfl7* [50,51]. Both *Egfl7* and *Mir126* are widely expressed in endothelial cells, and their close genomic localisation may facilitate this co-expression [50]. Two further examples are *Mir208a* and *Mir208b*, which are located in introns of *Myh6* and *Myh7* respectively and are differentially expressed in parallel with their host gene expression in mouse heart, suggesting that the miRNAs are processed from the intronic transcript rather than being transcribed as a separate RNA [40,41].

## 7 Conditional knockouts reveal global microRNA requirements

*Dicer* knockouts have also contributed to the association of microRNAs with disease by effectively knocking out all microRNAs in the targeted cells. Mice lacking *Dicer* expression in the Purkinje cells of the brain develop ataxia and degeneration of the Purkinje cells [52], and loss of *Dicer* in excitatory forebrain neurons leads to microcephaly among other neural defects [53]. Conditional knockout of *Dicer* in the podocytes of the kidney causes cytoskeletal disorganisation of the podocytes, resulting in progressive glomerulonephritis and death of the mutant mice by 6 weeks after birth [54]. Inactivation of *Dicer* in the retina, using a transgene which results in a mosaic pattern of *Dicer*-expressing cells next to *Dicer*-null cells, caused retinal degeneration [55]. Knocking out *Dicer* in the developing mouse liver results in hepatocyte proliferation and apoptosis, and promotes hepatocarcinogenesis [56], while loss of *Dicer* in the pancreas leads to a severe reduction in β cells [57]. Multiple reproductive defects and female sterility occurs in mice with *Dicer* deleted in the developing Müllerian duct [58]. Loss of *Dicer* in skin follicles from E14.5, which is when primary hair follicle development begins, results in dehydration after birth, probably due to the loss of epidermal barrier function. Most of the conditional knockout mice die by about postnatal day (P)6, but before death, whisker and hair follicles are clearly abnormal, with cysts appearing in the epidermis [59,60]. Conditional knockout in the hair follicles of *Dgcr8*, which is also involved in microRNA processing, results in a very similar phenotype, confirming that microRNAs are important for skin development [61]. Conditional *Dgcr8* elimination in cardiomyocytes led to dilated cardiomyopathy and early death [62].

Conditional knockouts of *Dicer* have been made to study the effects of total microRNA loss in the inner ear. When driven by *Pax2-Cre*, *Dicer* expression is lost in the otocyst, kidney and midbrain–hindbrain from about E8.5 [63]. The conditional *Dicer* knockout mice showed gross malformations of the inner ear, with loss or reduction of the lateral and anterior semicircular canals and cristae, which house some of the vestibular sensory hair cells. The cochlea is also misshapen, lacking coils and with only patches of sensory hair cells which fail to organise correctly into rows of inner and outer hair cells [64]. A hair-cell specific *Dicer* knockout has also been made, using *Pou4f3-Cre*, which drives expression in the cochlear and vestibular hair cells [65]. The resulting mice were deaf, and had a mild balance phenotype indicative of vestibular defects. At embryonic day 18.5 and at birth, the inner ear sensory epithelia looked unaffected, but by postnatal day 38, the hair cells showed signs of degeneration [66].

## 8 MicroRNAs in Mendelian disease

Despite demonstrations of the role of microRNAs in disease mechanisms, mutations leading to a Mendelian disease have been harder to find. Single nucleotide polymorphisms have been described in human microRNA precursor sequences but without any obvious association with disease [67,68]. Now, three single base pair changes in miR-96, two in humans and one in mouse, have been described associated with progressive hearing loss [23,69].

*Mir96*, *Mir182* and *Mir183* are located close to each other in an intergenic region on chromosome 6 in the mouse and are thought to be transcribed together. They are expressed in the sensory hair cells of the inner ear, both the inner and outer cochlear hair cells, which are located in the spiral organ of Corti and detect sound, and in the vestibular hair cells, which detect gravity and motion [70].

### 8.1 Diminuendo: a single base pair substitution in miR-96

**Fig. 2:**
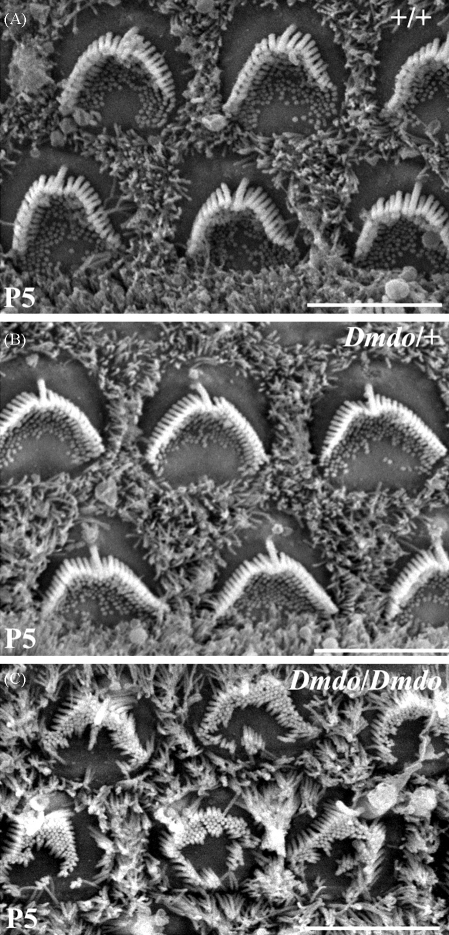
Scanning electron microscopy of diminuendo homozygote and heterozygote hair cells at 5 days old. (A–C) Outer hair cells of wildtype (A), heterozygote (B) and homozygote (C) mice at 5 days old. Heterozygote hair cells appear indistinguishable from wildtype hair cells at this early stage, but homozygote hair cells show ectopic stereocilia and disorganisation. Scale bars = 5 μm.

*N*-ethyl-*N*-nitrosurea (ENU) is a powerful mutagen that introduces single base pair mutations to the genome at random locations. In a screen for dominantly inherited deafness, the offspring of ENU-mutagenised males were tested for hearing impairment [71], and one of the resulting mice, presumably a heterozygote, demonstrated progressive hearing loss between 4 and 6 weeks old. Mice homozygous for the mutation, named diminuendo (*Dmdo*), were profoundly deaf and exhibited circling behaviour, indicating vestibular dysfunction. Scanning electron microscopy at 4 weeks old showed both outer and inner hair cells were misshapen and disorganised in the heterozygotes, while in homozygotes, hair cells had degenerated almost entirely. Earlier in development, homozygote hair cells were present but were abnormal in appearance, with ectopic stereocilia (Fig. 2) [23].

**Fig. 3:**
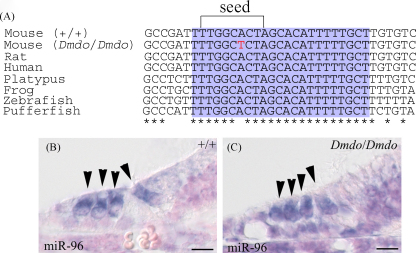
miR-96 conservation and expression. (A) Alignment of DNA sequences from wildtype mouse, diminuendo homozygote, rat, human, platypus, frog, zebrafish and pufferfish. The purple region shows the mature miRNA sequence for each species, and the bracket delineates the seed region critical for target binding. The mutation in the seed region is indicated by the red letter. The mature sequence is absolutely conserved between the species shown, and also between cow, dog, horse, macaque, opossum, chimpanzee, orang-utan, ground squirrel, tree shrew, mouse lemur, bushbaby, cat, armadillo, tenrec, medaka, rabbit, stickleback and tetraodon (sequences obtained from Ensembl v50; http://www.ensembl.org). (B and C) Expression of *Mir96* at P5 in wildtype (B) and homozygote (C). Hair cells are marked by arrowheads. Scale bars = 10 μm. Part A reprinted with permission from Lewis et al. [23].

The mutant phenotype was mapped to a 4.96 Mb interval on chromosome 6, and exons were sequenced from wildtype and homozygote DNA to locate the mutation. A single A > T base pair substitution was found in the seed region of *Mir96*, the region which is crucial for microRNA-target binding [72]. This nucleotide, and indeed, the entire microRNA sequence, is conserved between human, mouse, rat and fish (Fig. 3A). Expression of the mutant microRNA appears unaltered, at least spatially; miR-96, miR-182 and miR-183 are all detected in wildtype and diminuendo mutant hair cells during the first few days after birth (Fig. 3B and C) [23].

### 8.2 Effects of the diminuendo mutation on gene expression

**Fig. 4:**
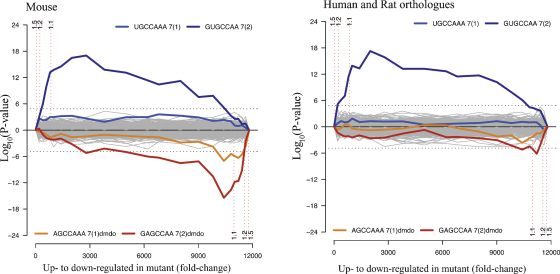
Hypergeometric analysis of microarray data comparing diminuendo homozygote to wildtype at 4 days old. (A) Microarray analysis showing enrichment and depletion of heptamers in 3′UTRs using Sylamer. The *x*-axis represents the sorted gene list from most upregulated (left) to most downregulated (right). The *y*-axis shows the hypergeometric significance for enrichment or depletion of heptamers in 3′UTRs of the genes under consideration when compared to the 3′UTRs in the complementary gene set. Positive values indicate enrichment (−log10(*P*-value)) and negative values depletion (log10(*P*-value)). Any heptamers which are present more often than would be expected will stand out above the significance line (adjusted *P*-value = 0.01), and any which are present less often than would be expected will stand out below the significance line on the negative axis. (B) The same analysis as in A, where each 3′UTR has been replaced by the concatenation of its orthologous 3′UTRs in human and rat. The seed match for the wildtype miR-96 shows similar enrichment as compared with the analysis in A. In contrast, the enrichment of the miR-96 diminuendo mutant binding sites in the downregulated genes is barely above background (dotted line). Reprinted with permission from Lewis et al. [23].

If the seed region is important for binding to the target sequence in a messenger RNA, such a mutation is likely to both lose repression of normal targets and gain repression of novel targets. In order to test this, a microarray was performed on RNA from the 4-day-old organ of Corti of homozygous mutants compared with wildtype controls, and 96 transcripts were found to be significantly affected. A Sylamer analysis [73] was performed on data from the entire microarray to detect any enrichment of heptamer sequences in transcripts organised in order of degree of up- or downregulation (Fig. 4). Among the upregulated genes, the heptamer complementary to the wildtype seed region was significantly enriched, and this enrichment was also observed when the analysis was run using the human orthologues of the mouse genes in the microarray, implying conservation, and therefore function. Among the downregulated genes, the heptamer complementary to the mutant seed region was enriched, but this enrichment was barely conserved with the human orthologues, which is to be expected, because the mutant site is not a naturally occurring functional sequence and hence is not likely to be conserved through selective pressure [23]. The results of this analysis demonstrate that (a) hundreds of genes are targeted by miR-96, (b) the mutant miR-96 targets new transcripts, and (c) miR-96 has an impact on mRNA levels in addition to any effects it may have on protein translation.

**Fig. 5:**
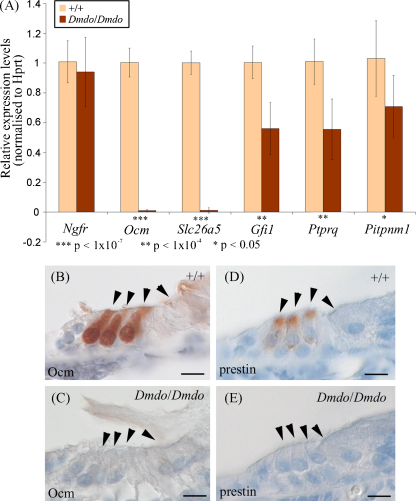
*Ocm*, *Slc26a5*, *Gfi1*, *Ptprq* and *Pitpnm1* in diminuendo. (A) Quantitative real-time PCR on cDNA generated from normalised RNA from the organs of Corti of 4-day-old littermates. *Ocm*, *Slc26a5* (prestin), *Ptprq*, *Pitpnm1* and *Gfi1* are downregulated in homozygotes. Error bars represent standard deviation. Quantities normalised to Hprt1 levels; Ngfr is expressed in support cells adjacent to hair cells [80] and was used to assess the quantity of sensory material. Three animals were used per genotype and DNA from each was run in triplicate. (B–E) Location of oncomodulin (B and C) and prestin (D and E) in 5-day-old wildtype (B and D) and homozygote (C and E) littermates. Scale bars = 10 μm.

Among the genes that were most downregulated in the diminuendo mutant inner ear were *Slc26a5* (prestin), *Ocm*, *Pitpnm1*, *Gfi1* and *Ptprq*. Changes in expression of these five genes were verified by RT-PCR and by immunohistochemistry (Fig. 5). All five are strongly and specifically expressed in the hair cells of wildtype mice, and *Ptprq*, *Gfi1* and *Slc26a5* are known to be important for development and function of hair cells because mice null for each gene exhibit hair cell degeneration and deafness [74–77]. The fact that all five genes were downregulated but do not bear the mutant target sequence means that they must be downstream of a cascade of regulation which is headed by miR-96. The primary target or targets of miR-96 in the inner ear have not yet been discovered [23].

The diminuendo mutation is the first ENU-induced mutation in a microRNA, and the first microRNA to be associated with deafness. miR-96 mutations have also been associated with progressive deafness in two human families [69]. The microRNA exerts its effects upon a range of genes, as seen by the Sylamer analysis, and the results of its action include marked downregulation of several genes important in hair cell development and function. As such, it is a prime example of microRNA function in both development and disease.

## 9 Conclusion

Since the first microRNA was identified 17 years ago, miRNAs have become an acknowledged part of the machinery of gene regulation, and their roles in multiple systems and cell types are still being discovered and characterised. Their importance is evident from the phenotypes of knockout and mutant mice, and the numerous studies comparing expression profiles offer an indication of just how many microRNAs may be required by each cell type for correct development and function. The mouse is an important organism for this work, and it is to be expected that future microRNA studies will offer even more insights into the intricacies of development, and hopefully new therapeutic targets for treating disease.
